# An Innovative App (ExoDont) for Postoperative Care of Patients After Tooth Extraction: Prototype Development and Testing Study

**DOI:** 10.2196/31852

**Published:** 2021-12-31

**Authors:** Meenakshi Krishna, Deborah Sybil, Priyanshu Kumar Shrivastava, Shubhangi Premchandani, Himanshu Kumar, Pintu Kumar

**Affiliations:** 1 Faculty of Dentistry Jamia Millia Islamia New Delhi India; 2 Department of Oral and Maxillofacial Surgery Faculty of Dentistry Jamia Millia Islamia New Delhi India; 3 WebDev Creations New Delhi India

**Keywords:** ExoDont, Android app, teledentistry, mHealth, tooth extraction, postoperative, dentistry, dentist, teeth, dental surgery, oral surgery

## Abstract

**Background:**

The postoperative period is crucial for the initiation of healing and prevention of complications after any surgical procedure. Due to factors such as poor compliance, comprehension, and retention of instructions, and other unaccounted factors, the objectives of postoperative care are not always achieved. Therefore, an Android-based mobile health app (ExoDont) was developed to ensure a smooth postoperative period for patients after a dental extraction. The ExoDont app delivers reminders for postoperative instructions and drug intake at defined intervals, thus fostering self-reliance among patients in taking their prescribed dose of medication.

**Objective:**

The aim of this study is to design, develop, and validate ExoDont, an innovative app for improved adherence to postoperative instructions after tooth extraction.

**Methods:**

A postoperative treatment protocol was developed by a team of oral and maxillofacial surgeons and general dentists, following which the clinical and technological requirements of the app were determined along with the software engineers, graphic designers, and applications architect in the team. ExoDont was developed to provide timely reminders for medication and postoperative care. The app was field tested and validated using the User Version of the Mobile Application Rating Scale.

**Results:**

The ExoDont software design was divided into a 3-level architecture comprising a user interface application, logical layer, and database layer. The software architecture consists of an Android-based ExoDont app for patients and a web version of the admin panel. The testing and validation of the ExoDont app revealed that Perceived Impact received the highest mean score of all rated components (mean 4.6, SD 0.5), while Engagement received the lowest mean score (mean 3.5, SD 0.8).

**Conclusions:**

The testing and validation of the app support its usability and functionality, as well as its impact on users. The ExoDont app has been designed, keeping the welfare of patients in view, in a user-friendly manner that will help patients adhere to the prescribed drug regimen and ensure easy and efficient dissemination of postoperative instructions. It could play an instrumental role in fostering compliance among patients and significantly decrease the complication rate following dental extractions.

## Introduction

One of the most important factors that influences the recovery process after any surgical procedure is adherence to postoperative care instructions. Patient compliance describes the degree to which a patient follows medication regimens and instructions given by the doctor [1]. A successful postoperative care period depends on the patient understanding and implementing the guidelines as advised by treating doctors to minimize morbidity and surgery-related complications, and to improve quality of life [2].

Tooth extraction is the most common surgical procedure in oral surgery. Compliance with postoperative instructions after a tooth extraction is influenced by language difficulty, low health literacy, inadequate surgeon-patient communication, and patient inability to concentrate on instructions due to postoperative stress and their emotional and psychological state [3-5].

The World Health Organization classifies the lack of adherence to posttreatment instructions as a major global problem [6]. Studies estimate that around 20%-50% of patients do not take their medication correctly [7-9] and the reasons for this nonadherence are varied, with the most frequent reason being that it was involuntary (ie, either confusion or forgetfulness). This highlights the need for designing a system and method that would foster adherence in patients and help reduce postoperative complications.

With this in mind, our team developed an innovative software application for timely delivery of postoperative instructions named ExoDont. ExoDont is an Android-based hybrid application aimed at fostering treatment adherence in patients undergoing tooth extractions. It is an attempt toward encouraging the public to take ownership over their medication use—including taking the prescribed dose at the right frequency and for the correct duration—with a personalized, easy-to-use innovative app-based system that displays reminders to take medication at appropriate times and illustrates postoperative instructions. We hypothesize that the use of this internet-based application will improve patient compliance and reduce complications after tooth extraction over the conventional mode of transmission of postoperative instructions; a detailed analysis will be presented as the second part of the project.

## Methods

### Developing a Postoperative Treatment Protocol

The first step in developing the ExoDont app was to establish the postoperative treatment protocol, which included antibiotics and analgesics along with postextraction instructions to be prescribed to the patient after tooth extraction. A team of oral and maxillofacial surgeons along with 2 general dentists developed a list of postprocedural medications (antibiotics, analgesics, multivitamins, antacids, etc) along with their dosages based on international recommendations [10]. A list of postoperative instructions was formulated by referring to previously available data [2].

### Determining Clinical and Technological Requirements

The technology team, consisting of software engineers, a graphic designer, and an applications architect, was then briefed about the app requirements. The following list of requirements was provided to the technology team: (1) the app should provide the abovementioned list of medications for the doctor to choose from, (2) patients should get timely instructions and reminders based on the time of tooth extraction and frequency and duration of prescribed medication, (3) the user interface should display the prescription and all instructions for the patient to view once logged into the app, and (4) patient data privacy should not be compromised.

### Determining App Design

The technological team laid out the app design considering the following points. First, the app should ensure the confidentiality of patient information. Second, the system needs to be user-friendly for both the admin/doctor and the patient. Third, there needs to be a back-end server that stores and manages notifications sent to the patient. Fourth, it should be usable and adaptable for common operating systems. The patient will be required to download the app on his/her Android-operated device and enter the user ID and password provided by the authorized individual who will enter the patient credentials as well as the prescribed medication via a secured web page.

### Field Testing and Validation of the App

Field testing was done in a group of 5 volunteers to check for the accuracy of the app in delivering the right instructions and reminders for medication. The app was tested in a group of 10 patients who underwent tooth extraction. The modified User Version of the Mobile Application Rating Scale (uMARS) was used to critically appraise the app.

## Results

### Overview

The ExoDont software system design was divided into a three-level architecture: (1) user interface application, (2) logical layer, and (3) database layer.

Both the user interface layer and logical layer are separate from the database layer. This design protects patient/user privacy and permits only authorized hospital staff to access the data from the admin interface. The design of the software relies on client-server architecture, with the user application interfaces operating as clients (resource and service requesters) and the back-end system operating as the server (resource and service provider).

### Client-Side Software

In the development of ExoDont, the Ionic framework was used, which is an open-source mobile user interface toolkit for building high-quality, cross-platform native and web app experiences; it is fast, has a single code base, and runs everywhere with JavaScript. The framework works with Angular, with TypeScript as the programming language.

### Server-Side Software

When designing the server-side software, data security and cross-platform connectivity were taken into consideration. Node.js with an Express backend was used in its development, which includes a support library that interacts with popular database management systems and protects against malicious attacks. The admin panel was designed to allow an authorized person to enter patient details and choose suitable medications and instructions to be given to the patient.

### Software Architecture

#### ExoDont

**Figure 1:**
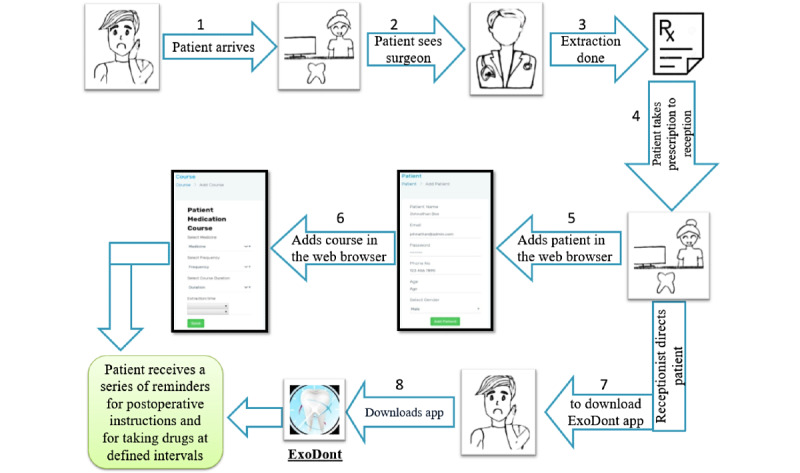
Workflow of the ExoDont app.

**Figure 2:**
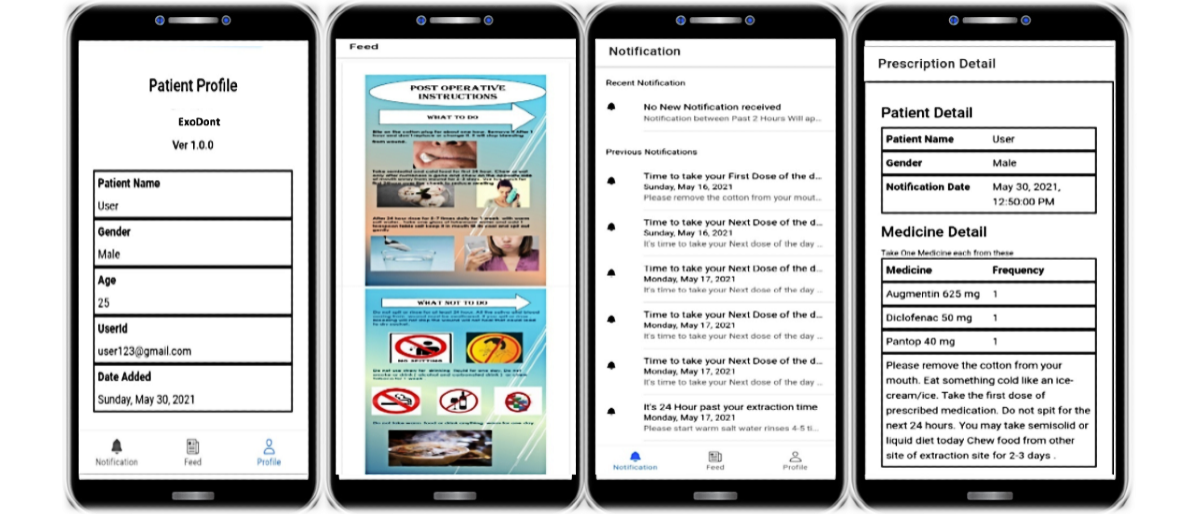
User interface of the mobile version of the ExoDont app.

This Android-based app has to be downloaded onto the patient’s mobile phone. The patient will be able to access it using the login credentials provided by an authorized person. The entire process at reception takes less than 10 minutes, after which the patient will be able to view the home screen displaying all current and past notifications (Figure 1). A feed section with oral health information and a profile page with the patient’s personal information will also be made available to the user. The interface is user-friendly and enables quick and simple input (Figure 2).

#### Admin Panel

**Figure 3:**
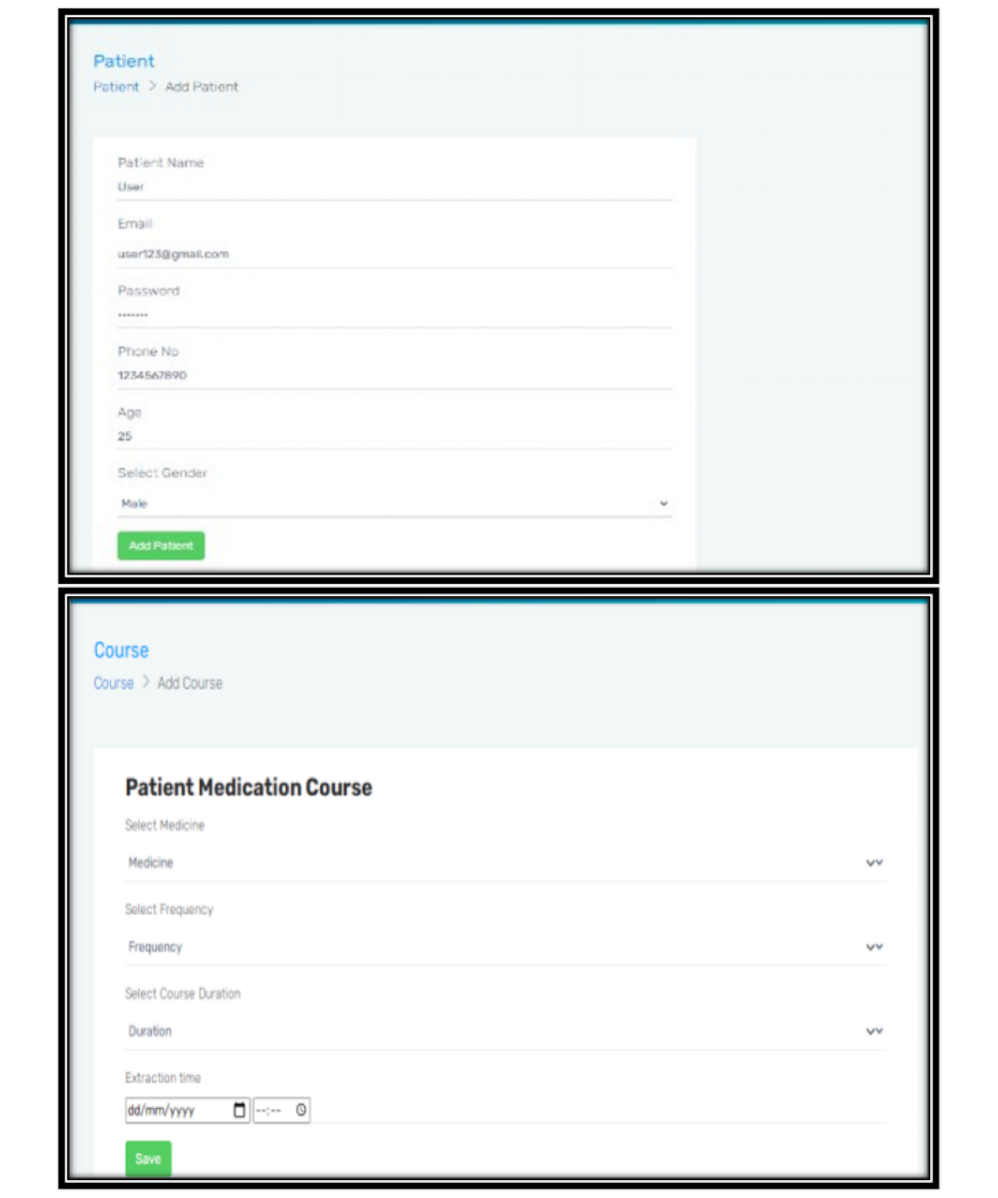
The web version of the ExoDont app.

Only an authorized admin/doctor was allowed access to this panel using login credentials made available exclusively to him/her. Once logged in, the admin was able to view all patient records and current prescriptions. He/she will be able to add a new patient, select required medications, and set up notifications according to the time of extraction (Figure 3). Nonproprietary (generic) names of drugs used in the prototype are available at all times in any region and the admin is free to choose from the list. This is independent of the market availability of that drug. Moreover, the list is customizable to address the specific needs of a particular region where drugs other than the ones mentioned may be in use.

### Database Implementation

To improve cross-platform connectivity, a popular and robust open-source database management system called MongoDB was used to capture, query, and administer the data collected by the ExoDont app. The database comprises the following 4 tables that store raw and processed data:

1. The patient table, which contains the personal information (name, age, gender, registration number) and login credentials of all patients.
2. The medicine course table, which includes details about the medicine course of all patients and gets updated with every new patient entered.
3. The medicine store table, which includes the list of all available medications the doctor can choose from.
4. The notification table, which contains information about all notifications sent to the patient and gets updated depending on the prescription of various patients.

### Testing and Validation

Field testing results showed that privacy of patient data was maintained in the app as no test participant could access details of other patients. The 10 patients who used the app were asked to complete the uMARS questionnaire [11]. According to the results, the Perceived Impact of the app received the highest mean score (4.6, SD 0.5), with the individual scores ranging from 3.5-5.0. Functionality and Information received equal mean scores of 4.5 (ranging from 2.3-5.0 for Functionality and 3.0-5.0 for Information). The lowest mean score was observed for Engagement (3.5, SD 0.8), with an individual score range of 1.8-4.8. The detailed uMARS results are presented in Table 1.

**Table 1:** Mobile App Rating Scale results for the ExoDont app.

Participant	Engagement	Functionality	Esthetics	Information	Subjective quality	Perceived impact	Overall mean (per participant)
1	1.8	2.3	2.0	3.0	2.8	3.5	2.6
2	3.6	5.0	4.0	4.8	4.3	4.7	4.4
3	4.8	5.0	4.7	5.0	4.5	5.0	4.8
4	4.2	5.0	4.0	4.5	3.8	4.5	4.3
5	3.4	4.5	3.7	5.0	4.0	5.0	4.3
6	3.6	4.5	4.7	4.8	3.5	4.3	4.2
7	3.0	4.5	3.0	4.5	3.3	4.5	3.8
8	3.4	5.0	4.3	5.0	4.5	5.0	4.5
9	3.2	5.0	3.3	4.0	3.3	4.3	3.9
10	3.8	4.3	4.3	4.5	3.5	4.7	4.2
Overall mean (SD) by component	3.5 (0.8)	4.5 (0.8)	3.8 (0.8)	4.5 (0.6)	3.7 (0.6)	4.6 (0.5)	4.0 (0.6)

## Discussion

### Principal Findings

The past few years have seen a greater reach of mobile devices in developing countries such as India, much more than that of the necessities of electricity, roads, and clean water. Extensive and rapid development of mobile technology, a fall in market prices of products, and a large increase in rates of use are the driving factors of the increase in eHealth delivery systems [12]. The vast array of smartphones, mobile tablets, and mobile medical apps has revolutionized healthcare delivery systems and has presented an unprecedented opportunity to consumers to achieve their healthcare goals [13]. Presently, there are more than 165,500 smartphone apps specifically related to health services, and one in five people have downloaded such mHealth apps [14]. The Clinical Event Annotator app for real-time patient monitoring [15]; the Mozzify app, featuring Dengue fever case reporting, a mapping system, and behavioral modification through reminders [16]; and the See Me Smoke-Free app [17] for smoking cessation, eating a healthy diet, and increasing physical activity are just a few of the many mHealth apps that tackle lifestyle issues in an efficient manner. Such apps have turned mobile devices into personal laboratories that have the potential to consistently assess a person’s physiology, behavior, social context, and environmental exposure [18].

There are also certain mobile healthcare apps available that support patients in adhering to their prescribed medication regimen through reminder functionalities. Medication adherence–based mHealth apps aim at delivering a behavioral intervention through reminder systems in the form of push notifications, text messages, text messages requiring a response, and other methods [19]. RxmindMe is a straightforward reminder-based app that informs the patient when the dose is due and additionally has a provision for recording when the dose was taken [20]. Another novel and advanced system based on this principle is SmartTrack, aimed at improving patient adherence to inhaler devices. It works by clipping onto inhaler devices and recording the date and time the inhaler was used; it also sends alerts when a defined dose is missed [21]. Positive outcomes in therapeutic adherence have been reported in multiple studies with a significant statistical difference in adherence before and after the introduction of mHealth interventions [22-24].

The emergence of technology-associated healthcare has paved the way for teledentistry, which includes a plethora of dental health apps that are being used for teleconsultation for oral health–related issues, booking appointments, and for electronic recordkeeping. The earliest evidence reported for online dental services was for teleassistance for minor orthodontic emergencies using mobile phones with a video function [25]. Since then, several other dental apps have been developed to cater to people’s oral health needs. One such app, DDS GP, provides patient information and treatment planning, while another app, BrushDJ, encourages the user to brush his/her teeth for the recommended time of 2 minutes using music as a timer. The latter also has pop-ups to remind the user to floss or change his/her toothbrush. Several other dental health apps, including Dental Monitoring, Dentists for me, and DDS Anywhere, have been well received by users, especially during the COVID-19 pandemic for emergency consultations across many specialties of the dental field [26]. However, none of these apps have addressed the issue of compliance and dental treatment adherence in patients for postoperative oral care. Furthermore, it has been found that the involvement of health care professionals in the process of app development is more likely to provide greater insight into patient needs and is indicative of more reliable content and higher quality, but many currently available mHealth apps are lacking in this area [27]. ExoDont is a unique app that provides an innovative approach to foster patients’ adherence to postoperative instructions and the prescribed medical regimen after tooth extraction. Furthermore, since ExoDont was developed in close association with health care professionals, it ensures better quality evidence regarding postoperative instructions and drug prescriptions. The patient will receive timely notifications reminding him/her to take the prescribed antibiotics. Completion of the antibiotic course is expected to reduce the incidence of antibiotic resistance and also decrease the likelihood of postoperative complications.

Common complications after tooth extraction include alveolar osteitis (also known as dry socket), infection, bleeding, and paresthesia [2,28]. To ensure healing of the surgical site, it is important that patients comply with postoperative instructions, which are traditionally given verbally or in writing. In a study conducted by Vallerand et al [3], it was found that compliance improved when patients were provided with both verbal and written instructions after third molar removal. However, Kessels [5] reported that patients forgot 40%-80% of the information given by the surgeon almost immediately. Similarly, Houts et al [29] stated that patients recalled only 14% of instructions given verbally as compared to 80% of instructions combined with pictograms. Conversely, in a study conducted by Alvira et al [2], no statistical difference in terms of adherence to postoperative instructions was observed with regard to the manner in which information was presented. ExoDont has thus been introduced to provide a definitive solution. It is expected to increase patients’ adherence to the prescribed medication regimen and postprocedural instructions and in turn reduce postoperative complications and enhance healing. By providing prompt reminders, the app is expected to help circumvent forgetfulness. A study by Vettori et al [30] was able to establish a positive correlation between the occurrence of alveolar osteitis and a lack of compliance toward instructions such as refraining from smoking or using mouthwash.

Antibiotic dose optimization is crucial for any antimicrobial treatment or prophylactic regimen to be successful. For a drug to be effective against a microbe, it is essential that the drug remains at the site of infection at the optimal concentration and for an adequate amount of time. A drug should be able to produce concentration-dependent inhibition (by attaining a peak concentration with respect to the minimum inhibitory concentration) or time-dependent inhibition (by remaining at a concentration above the minimum inhibitory concentration for an adequate length of time). Any variation in the temporal spacing of the dose or noncompliance can cause a disturbance in the aforementioned factors and disturb the microbial flora, which may encourage the development of antimicrobial resistance [31]. The World Health Organization has declared antimicrobial resistance to be one of the top 10 global public threats facing humanity [32]. Dentists prescribe around 7%-11% of all the common antibiotics for oro-dental infections and the main cause of the development of antimicrobial resistance is the incorrect use of the same [33]. ExoDont aims to address these factors by promoting the appropriate use of antibiotics, thus reducing the likelihood of short courses, missed doses, or self-prescribed antibiotic intake in patients.

The critical appraisal of the ExoDont app using the uMARS scale suggested that the participants found the app to be lagging behind in engagement, which can be justified as per the aims and objectives of the app. The app was designed solely for the purpose of sending reminders and postoperative care instructions to patients, without any scope for customization or feedback on their part, which explains why this aspect of the app received the lowest score. The highest scores were awarded to the app’s Functionality and Perceived Impact. The app aims at improving postoperative care through its functionality, as there is currently a lack of conclusive evidence regarding the best method of dissemination of postoperative instructions. The perceived impact rating of the app suggests that the app will be successful in achieving its objectives. Esthetics and Subjective Quality are aspects of the app that will be improved in future modified versions.

**Figure 4:**
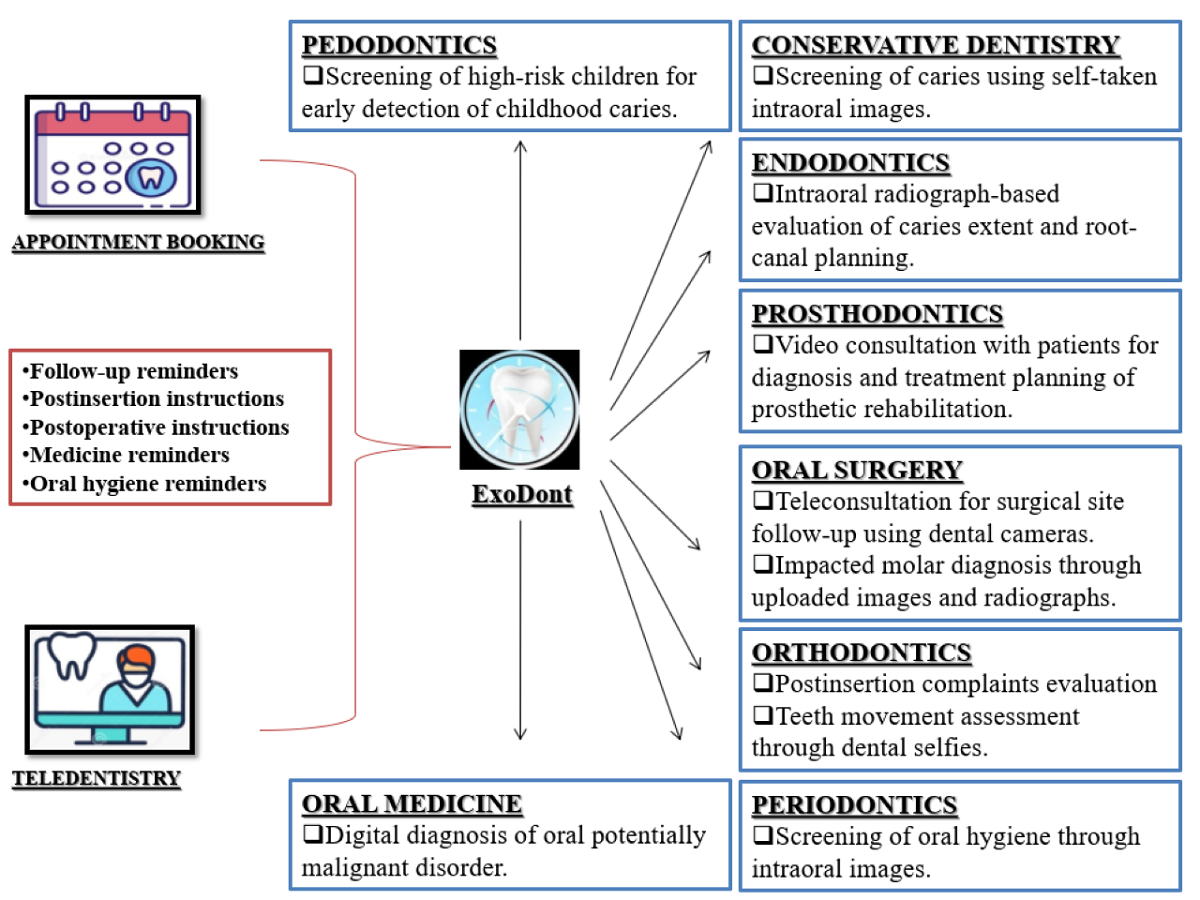
Reproducibility and application of the ExoDont app.

ExoDont can be thought of as a first step toward an even broader and holistic idea that includes all specialties of the dental field. This prototype presents an innovative scope to teledentistry and can be expanded to allow a host of other multidisciplinary functions too. From booking appointments to providing teleconsultation, ExoDont can be broadened in scope to include all specialty-specific functions. Figure 4 describes the broader scope of potential future applications within ExoDont. With the inclusion of multiple specialties and tasks that the app can perform, preventable complications can be reduced to a greater extent. It is expected that patients would have access to solutions to various oral health issues within a single app. ExoDont is also a sustainable solution in light of the COVID-19 pandemic. It could be helpful for providing access to oral healthcare remotely while a patient is at home.

As with any technological advancement, ExoDont too has certain limitations. The app is useful only in the subset of the population that uses smartphones. An internet connection must be maintained at all times during the postoperative period for timely notifications and prompts through the app. The field testing of the app involved a smaller group of patients to check for any major functional flaws of the app. Therefore, the limited responses may not be a very accurate representation of a broader group’s rating of the app. As a second part of this study, the app will be tested on specific parameters clinically with a greater sample size; it will be compared with the existing modalities of postoperative instruction dissemination. Notably, ExoDont is an Android app and it is not currently available to iOS users. Furthermore, the app does not take into account educational barriers and health literacy, which may negatively impact the usability of the app.

### Conclusions

ExoDont is a promising mHealth app specifically addressing postoperative treatment and medication adherence after tooth extraction. The app is expected to show improved patient compliance and increased medication adherence. Initial studies have demonstrated acceptability and ease of use by both the dentist and patient. Future studies are required to establish the advantages of this app over the conventional mode of postoperative care. Therefore, as a second part to this study, the ExoDont app will be tested against the conventional modes of postoperative care on parameters such as adherence to medication, functionality of the ExoDont app in a larger sample size, and reduction in postoperative complication rate.

## Abbreviations

uMARS: User Version of the Mobile Application Rating Scale
